# The degree of astrocyte activation is predictive of the incubation time to prion disease

**DOI:** 10.1186/s40478-021-01192-9

**Published:** 2021-05-12

**Authors:** Natallia Makarava, Olga Mychko, Jennifer Chen-Yu Chang, Kara Molesworth, Ilia V. Baskakov

**Affiliations:** 1 Center for Biomedical Engineering and Technology, University of Maryland School of Medicine, 111 S. Penn St, Baltimore, MD 21201 USA; 2 Department of Anatomy and Neurobiology, University of Maryland School of Medicine, Baltimore, MD 21201 USA

**Keywords:** Prion, Prion diseases, Reactive astrocytes, Neuroinflammation, Prion strains, Neurodegenerative diseases

## Abstract

In neurodegenerative diseases including Alzheimer’s, Parkinson’s and prion diseases, astrocytes acquire disease-associated reactive phenotypes. With growing appreciation of their role in chronic neurodegeneration, the questions whether astrocytes lose their ability to perform homeostatic functions in the reactive states and whether the reactive phenotypes are neurotoxic or neuroprotective remain unsettled. The current work examined region-specific changes in expression of genes, which report on astrocyte physiological functions and their reactive states, in C57Black/6J mice challenged with four prion strains via two inoculation routes. Unexpectedly, strong reverse correlation between the incubation time to the diseases and the degree of astrocyte activation along with disturbance in functional pathways was observed. The animal groups with the most severe astrocyte response and degree of activation showed the most rapid disease progression. The degree of activation tightly intertwined with the global transformation of the homeostatic state, characterized by disturbances in multiple gene sets responsible for normal physiological functions producing a neurotoxic, reactive phenotype as a net result. The neurotoxic reactive phenotype exhibited a universal gene signature regardless of the prion strain. The current work suggests that the degree of astrocyte activation along with the disturbance in their physiological pathways contribute to the faster progression of disease and perhaps even drive prion pathogenesis.

**Supplementary Information:**

The online version contains supplementary material available at 10.1186/s40478-021-01192-9.

## Introduction

Transformation of astrocytes into reactive states is recognized as one of the major hallmarks of neurodegenerative diseases including Alzheimer’s, Parkinson’s, Amyotrophic Lateral Sclerosis and prion diseases [1, 2]. The role of reactive astrocytes in neurodegenerative diseases in not well understood. The questions whether reactive astrocytes are neurotoxic or neuroprotective, and whether their normal physiological functions are undermined remain unsettled [3–5]. Another important question is whether animal models faithfully reproduce glial phenotypes associated with neurodegenerative diseases in humans [6–8]. Unlike other neurodegenerative diseases, which rely on modeling of pathological condition in genetically modified animals, wild type or inbred animals infected with prions develops bona fide prion diseases [9–11]. Animals infected with prions offer an opportunity to examine the role of glia in actual chronic neurodegenerative disease, not a disease model.

Several recent studies suggested that astrocytes are intimately involved in prion disease pathogenesis, yet their role remains poorly defined [12–16]. In prion diseases, astrocytes responded to prion infection much earlier than neurons, and perhaps even prior to microglia [13]. Changes in astrocyte function were found to score at the top of the pathways activated in prion diseases [13]. Inhibition of PERK-elF2a signaling selectively in astrocytes was found to delay progression of prion diseases in mice [14]. Unexpectedly, elimination of three microglia-derived factors TNF-α, IL-1α and C1qa, which were thought to drive polarization of astrocytes into a neurotoxic state, accelerated the progression of prion diseases [12]. Moreover, partial depletion of microglia exacerbated the reactive phenotype of astrocytes and accelerated disease progression [16]. Highly infectious prions purified from animals were not directly neurotoxic, supporting the hypothesis on a non-autonomous mechanism being behind prion neurotoxicity [17]. Indeed, reactive astrocytes isolated from prion-infected animals exhibited synaptotoxic phenotypes characterized by impairment of neuronal growth, inhibition of dendritic spine development and synapse maturation along with impairment of synapse integrity [15].

The current study examined region-specific changes in astrocyte function and their reactive phenotype by monitoring the expression of astrocyte-specific genes along with markers of astrocyte reactive states in animals challenged with four prion stains via two different routes. One can expect the most severe changes in the degree of astrocyte activation and intensity of their response occur in animal groups with long incubation times to disease due to longer exposure to altered brain homeostasis. Instead, strong reverse correlation between the degree of astrocyte activation and the incubation time to terminal diseases was observed. Reactive states associated with prion diseases involved global transformation of the physiological functions, characterized by disturbance in multiple physiological pathways. The current work illustrated that the degree of astrocyte activation along with loss of normal homeostatic functions is predictive of the incubation time to the diseases, and suggests that these changes perhaps even drive prion disease progression.

## Materials and methods

### Animal experiments and brain tissue collection

Using isoflurane anesthesia, 6-week-old C57Black/6J female and male mice were inoculated with 10% or 1% brain homogenates, as indicated, prepared in PBS (pH 7.4) using terminally ill 22L-, ME7-, RML-, or SSLOW-infected mice. Brain materials from the 5th passage of SSLOW was used for inoculations [18, 19]. Inoculation volume was 200 μl and 20 μl for intraperitoneal (i.p.) and intracerebral (i.c.) inoculations, respectively. 1xPBS was inoculated into i.p. control groups. 10% brain homogenate prepared in PBS (pH 7.4) from healthy non-infected mice was inoculated into the i.c. control group. Animals were regularly observed for signs of neurological impairment: abnormal gate, hind limb clasping, lethargy, and weight loss. Mice were considered terminally ill when they were unable to rear and/or lost 20% of their weight. At this time, mice were euthanized by CO_2_ asphyxiation and their brains were immediately extracted. The brains were kept ice-cold for prompt dissection or preserved in 10% buffered formalin (MilliporeSigma) for histopathology.

Extracted ice-cold brains were dissected using a rodent brain slicer matrix (Zivic Instruments, Pittsburg, PA). A 2 mm central coronal section of each brain was used to collect individual regions. Allen Brain Atlas digital portal (http://mouse.brain-map.org/static/atlas) was used as a reference. The hypothalami (HTh), as well as thalami (Th), hippocampi (Hp) and cortices (Ctx) were collected into RNase-free, sterile tubes, frozen in liquid nitrogen and stored at −80˚C until the RNA isolation with Aurum Total RNA Mini Kit (Bio-Rad, Hercules, CA, USA), as described [13].

### Design of NanoString nCounter mouse astrocyte panel

To design custom-based nCounter Mouse Astrocyte Panel (Additional file 1: Table S1), publicly accessible databases (www.brainrnaseq.org and https://www.networkglia.eu/en/astrocyte) were used for selecting 275 genes that under normal conditions, express primarily in astrocytes and were assigned to functional pathways. In addition, 47 genes, reporting on reactive phenotypes, including A1-, A2- and pan-specific makers along with other markers of reactive astrocytes were used for the panel, as well as 8 microglia-, 10 neuron-, and 2 oligodendrocyte-specific genes. The addition of 10 housekeeping genes completed the panel bringing total number of genes to 352. These genes were assigned to 23 gene sets to allow for an advanced analysis of their group changes.

### Analysis of gene expression by NanoString

Samples were processed by the Institute for Genome Sciences at the University of Maryland, School of Medicine using a custom nCounter Mouse Astrocyte Panel. Only samples with an RNA integrity number RIN > 7.2 were used for Nanostring. All data passed QC, with no imaging, binding, positive control, or CodeSet content normalization flags. The analysis of data was performed using nSolver Analysis Software 4.0, including nCounter Advanced Analysis (version 2.0.115). For agglomerative clusters and heat maps, genes with less than 10% of samples above 20 counts were excluded. Z-score transformation was performed for genes. Clustering was done using Euclidian distance and the linkage method was Average.

For Fig. 2b and c, the number of differentially expressed genes (DEGs) was calculated from the ratio of normalized counts for each experimental group (*n* = 3) against the control normal i.p. female (F) group (*n* = 6). Only genes with *p* < 0.05 and linear fold change ≥  ± 1.2 were counted. Differential expression analysis for Fig. 3b and Additional file 7: Figures S3 and S4 was performed with nCounter Advanced Analysis. Only genes with *p* < 0.05 and linear fold change ≥  ± 1.2 were counted. Advanced Analysis was performed for each brain region separately. Undirected global significance scores obtained as a result of gene set analysis (GSA scores) were added up to produce a combined GSA score (a sum of GSA scores for all gene sets within one brain region) for plots on Additional file 7: Fig. S2. A sum of combined GSA scores for all four regions was used for plots on Fig. 4.

### Histopathology and immunofluorescence

Formalin-fixed brains were treated for 1 h with 95% formic acid to deactivate prion infectivity before being embedded in paraffin. Subsequently, 4 µm brain sections produced using a Leica RM2235 microtome were mounted on slides and processed for immunohistochemistry. To expose epitopes, slides were subjected to 20 min of hydrated autoclaving at 121 °C in Antigen Retriever citrate buffer, pH 6.0 (C9999, MilliporeSigma). Chicken polyclonal anti-GFAP (AB5541, MilliporeSigma) was used to stain for astrocytes. Detection was performed with a DAB Substrate Kit (BD Biosciences).

For co-immunofluorescence, rabbit polyclonal anti-AQP4 antibody (#HPA014784, Sigma-Aldrich) and chicken polyclonal anti-VIM (#ab24525, Abcam) were used in combination with chicken polyclonal anti-GFAP (AB5541, MilliporeSigma) or rabbit anti-GFAP clone D1F4Q (12,389, Cell Signaling Technology), respectively. The secondary antibodies were goat anti–rabbit or anti-chicken IgG conjugated with Alexa Fluor 546 for red color or Alexa Fluor 488 for green color (Thermo Fisher Scientific). Images were collected with an inverted microscope (Nikon Eclipse TE2000-U) equipped with an illumination system X-cite 120 (EXFO Photonics Solutions Inc., Exton, PA, United States) and a cooled 12-bit CoolSnap HQ CCD camera (Photometrics, Tucson, AZ, United States). Fiji ImageJ software was used for image analysis.

AQP4 distribution was measured on the images taken with 20× objective. 50 pixel long, linear profiles across cortical microvessels were exported to Microsoft Excel. The lines were drawn avoiding cell bodies, positioning the vessel in the middle. Measurements were performed on the brains of 4 infected animals and 3 control animals (3 fields of view for each brain, 10 measurements from each field of view, resulting in a total of 120 and 90 representative profiles from the infected and control animals, respectively). The minimum value of each profile was subtracted as a background value. Non-perivascular AQP4 signal was reported as an average intensity of the first and last 15 pixels of each profile. Maximum intensity of the 20 pixels in the middle of each plot represented the perivascular AQP4 signal. The ratio of perivascular AQP4 signal to the non-perivascular signal for each brain was plotted, and the statistical significance of the difference between infected and normal brains was estimated by an unpaired *t* test (****p < 0.0001).

### Statistics

In Figs. 2, 4, 5 and 6, Additional file 7: S2 and S3, correlation coefficients R^2^ and *p-*values were calculated using simple linear regression analysis performed with GraphPad Prism 9.0.0. In Fig. 8, the ratio of perivascular AQP4 signal to non-perivascular signal for each brain was plotted, and the statistical significance of the difference between infected and normal brains was estimated by an unpaired t-test (****p < 0.0001).

## Results

### Astrocyte panel detects region- and strain-specific differences in the gene expression profiles

For the analysis of gene expression, 352 genes were selected using publicly accessible databases (www.brainrnaseq.org and https://www.networkglia.eu/en/astrocyte) (Additional file 1: Table S1). The panel consisted of 275 genes, which were previously found to report on astrocyte physiological functions. An additional 47 genes were selected to report on reactive phenotype including A1-, A2- and pan-specific makers along with other markers of reactive astrocytes, or because of involvement of the genes in regulating astrocyte reactivity. The later groups of genes were used to assess the degree of astrocyte activation. The panel also included 8 microglia-, 10 neuron-, and 2 oligodendrocyte-specific genes along with 10 housekeeping genes. On average, the designed probes were positive for 99.9% of the samples, with 98.8% of probes generating a signal above 30 counts.

**Fig. 1:**
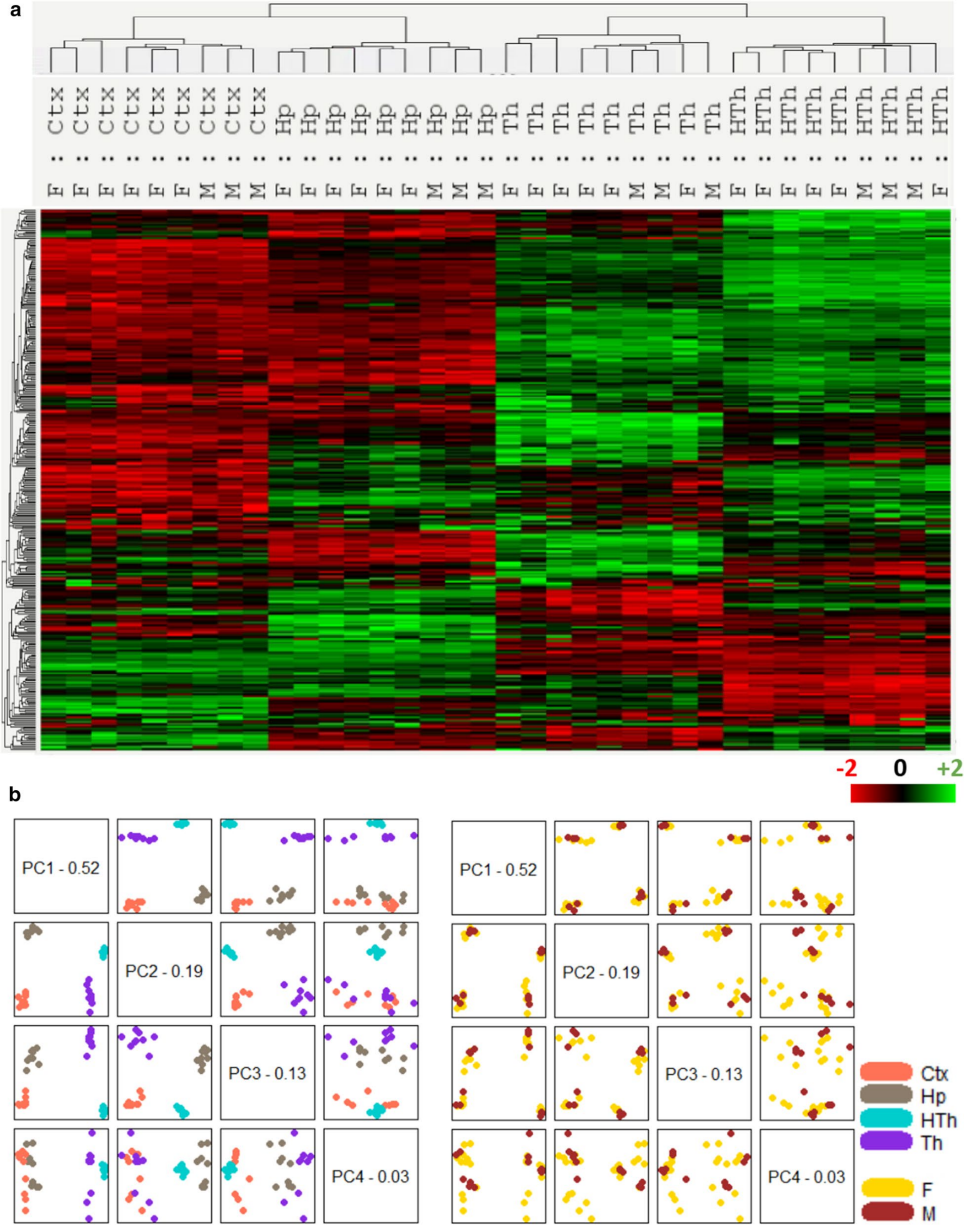
nSolver analysis of non-infected samples. (**a**) Hierarchical clustering of non-infected samples used as control groups showing high regional specificity and reproducibility of the Astorocyte panel. Adult C57Black/6J female (*n* = 6) and male (*n* = 3) mice were inoculated i.p. with PBS and analyzed at 197–363 days post-inoculation (Additional file 2: Table S2). (**b**) Principal component analysis illustrates strong differences in the expression pattern of brain regions (left panel) and no gender-specific differences (right panel). Th, thalamus; HTh, hypothalamus; Ctx, cortex; Hp, hippocampus; F, females; M, males

Based on previous studies [13], four brain regions—thalamus (Th), hypothalamus (HTh), cortex (Ctx) and hippocampus (Hp) were selected for examining region-specific gene expression. When non-infected adult C57Black/6J female (*n* = 6) and male (*n* = 3) mice were assessed by the astrocytic panel (Additional file 2: Table S2), the heat map of brain regions showed robust reproducibility and high regional specificity of the gene expression pattern (Fig. 1a). All samples clustered in strict accordance to brain region showing minimal variations within each control group. These results were consistent with previously reported well-defined region-specific homeostatic identities of astrocytes [13, 20–23]. Principal component analysis of non-infected samples confirmed strong region specificity, with very minimal, if any, gender-specific differences (Fig. 1b).

**Fig. 2:**
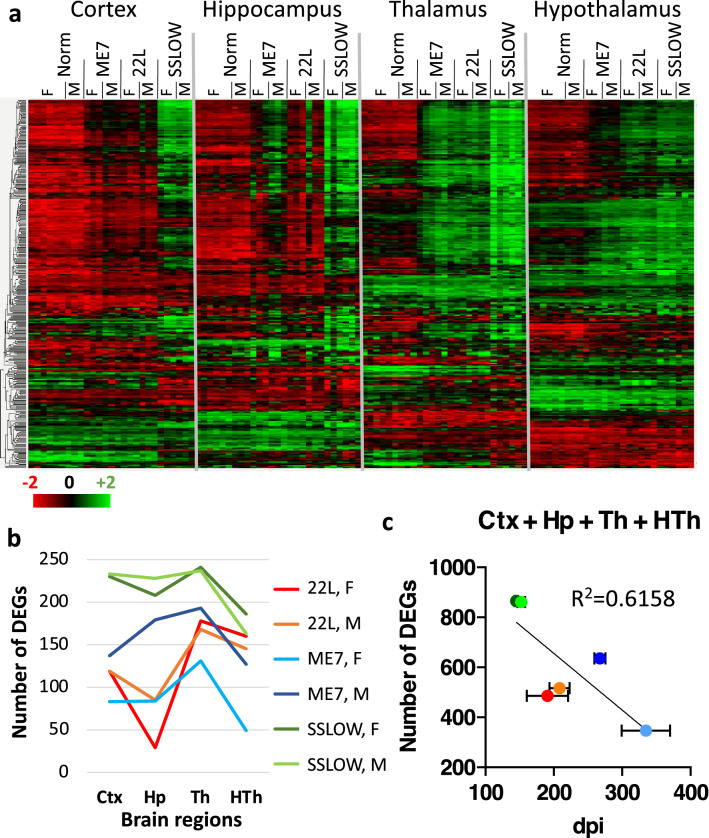
Region-specific response of astrocytes to prions in animals infected with ME7, 22L or SSLOW. (**a**) Ordered heat map of individual samples from animals inoculated i.p with ME7, 22L or SSLOW and control groups. (**b**) Strain profiles for female and male groups inoculated i.p. with 22L, ME7, or SSLOW. The profiles were built using the number of DEGs (linear fold change ≥  ± 1.2; *n* = 3; *p* < 0.05) in cortex, hippocampus, thalamus and hypothalamus. (**c**) Inverse correlation between the sum of DEGs in all four regions and the time to disease (dpi) in female and male mice inoculated i.p. with 22L, ME7, or SSLOW (*n* = 3). Bars represent the standard deviation in the time to terminal disease (*n* = 3). Th, thalamus; HTh, hypothalamus; Ctx, cortex; Hp, hippocampus; F, females; M, males

For examining the changes associated with prion diseases, C57Black/6J mice were inoculated intraperitoneally (i.p.) with 22L, ME7 or SSLOW mouse-adapted prion strains and analyzed at the terminal stage of the disease (Additional file 2: Table S2). Normalized counts for all genes in individual animals and fold change for each gene in four brain regions are listed in Additional file 3: Table S3 and Additional file 4: Table S4, respectively. Among three strains, SSLOW elicited the strongest response across four brain regions, whereas 22L and ME7 displayed noticeable region-specific tropism (Fig. 2a). These results were in agreement with previous studies that documented wide-spread neuroinflammation in SSLOW-infected animals [19], and strong regional tropism in 22L and ME7 animals [24, 25]. The number of genes differentially expressed (DEGs) in each region (p < 0.05, fold change ≥  ± 1.2) was used to build strain-specific response profiles (Fig. 2b). Because ME7 mice displayed strong gender differences in incubation time to terminal disease (Additional file 2: Table S2), male and female groups were analyzed separately for all strains. Surprisingly, the total number of DEGs that responded to prions in four regions correlated inversely with the time to the terminal stage of the disease (Fig. 2c). Staining with GFAP confirmed the region-specific and strain-specific differences in the degree of astrocyte activation (Additional file 7: Fig. S1). To summarize, in prion-infected brains, astrocyte reacted in a region-specific manner, where the degree of activation was dictated by the strain-specific tropism to individual regions (Fig. 2a). Nevertheless, upon summation of DEGs across four brain regions, strong reverse correlation (R^2^ = 0.61) between the total number of DEGs and incubation time to disease emerged.

### Inverse correlation between incubation time to the disease and degree of astrocyte response

As evident from a gradual increase in a number of DEGs during the course of the disease, progression of prion disease follows a strict strain-specific timeline [13, 24, 26, 27]. This observation holds true within each individual strain. However, comparison across strains at the terminal point revealed a reverse correlation: the group with the longest incubation time (ME7 females) displayed the least change in gene expression and vice versa (Fig. 2). This correlation is counterintuitive, because astrocytes in strains with longer incubation times were expected to have more severe changes due to longer exposure to altered brain homeostasis and more pronounced impact of natural aging.

**Fig. 3:**
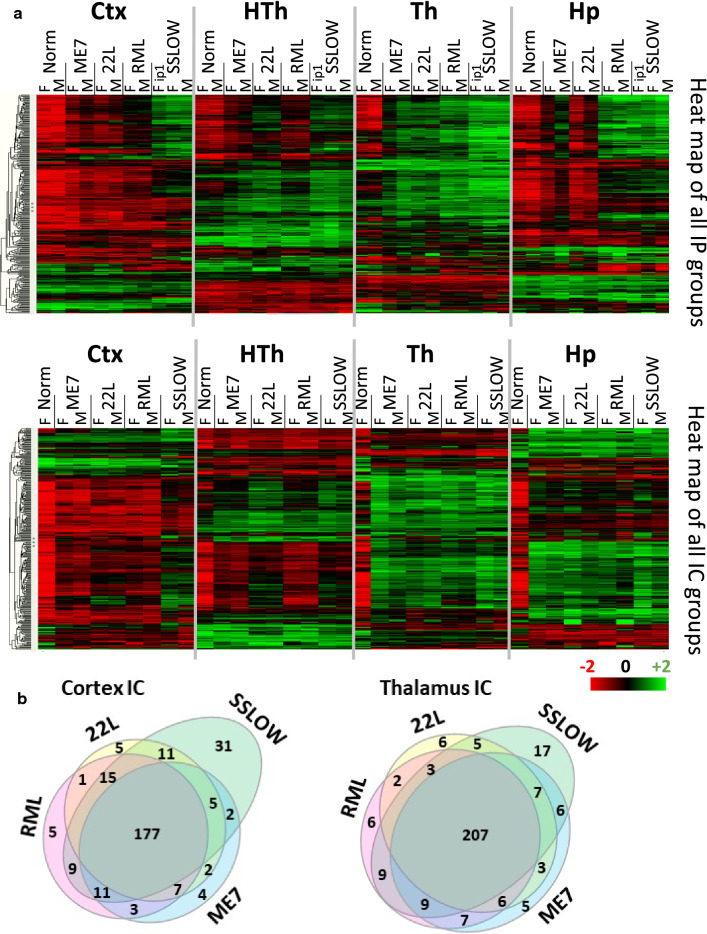
Changes in gene expression profiles detected with the Astrocyte panel in prion-infected mice. (**a**) Ordered heat map of grouped samples for cortex (Ctx), hypothalamus (HTh), thalamus (Th) and hippocampus (Hp) of normal and terminally ill females (F) and males (M) inoculated via i.p. (upper panel) or i.c. (lower panel) with ME7, 22L, RML, or SSLOW, as described in Materials and Methods. (**b**) Venn diagram for DEGs (linear fold change ≥  ± 1.2; *n* = 3; *p* < 0.05) in cortex (left) and thalamus (right) of i.c. inoculated ME7, 22L, RML, or SSLOW mice (females and males, *n* = 3 + 3)

For rigorously testing the relationship between the degree of changes in the astrocyte gene expression and incubation time, several groups were added to the analysis: animals challenged via intracranial route (i.c.) with SSLOW, 22L, RML or ME7; animals challenged via i.p. with RML; and an additional SSLOW group inoculated via i.p. (Additional file 2: Table S2). To summarize, female and male mice challenged with four prion strains via two routes were included into analysis (Fig. 3). To take into account a number of DEGs, but also weighted differences in the fold change and statistical significance, we used Gene Set Analysis (GSA) scores, which represented undirected global significance scores calculated by nCounter Advanced Analysis for each set of genes.

**Fig. 4:**
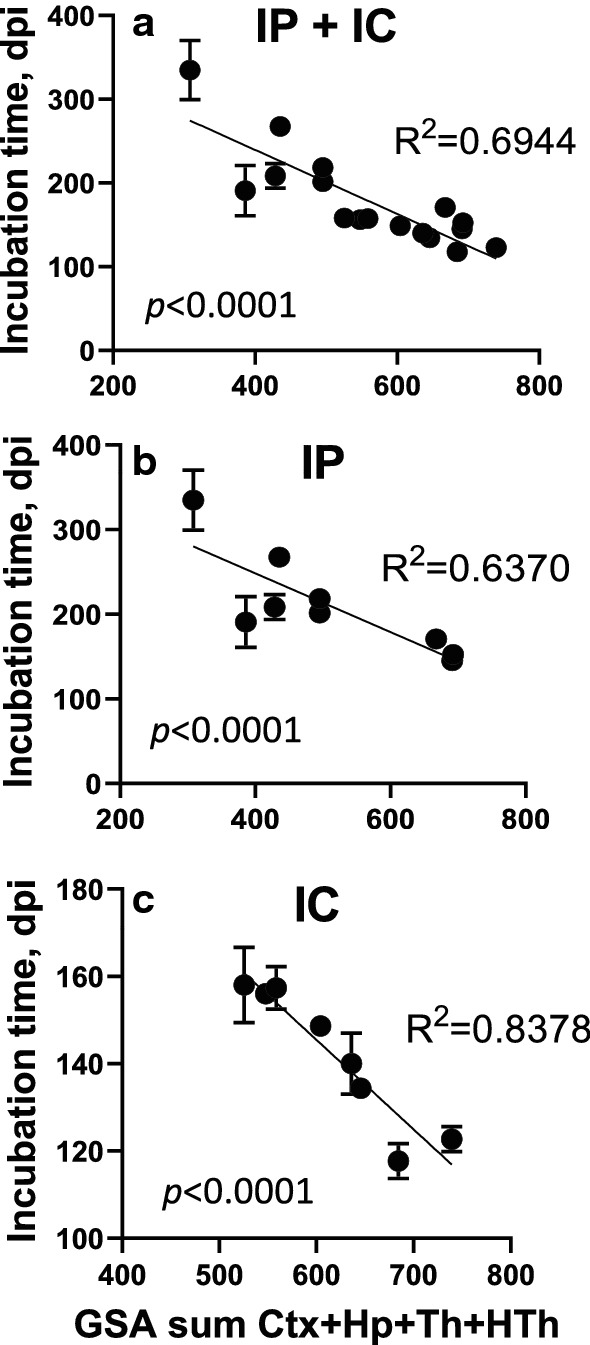
Inverse correlation between the sum of the combined GSA scores and the time to prion disease. A significant inverse correlation between the combined GSA scores summed across four brain regions and the time to terminal prion disease (dpi) in female and male mice inoculated with RML, 22L, ME7, or SSLOW (*n* = 3) shown for all sample groups i.p + i.c. (**a**), as well as separately for the i.p (**b**) and i.c. (**c**) samples. Bars represent the standard deviation in the time to terminal disease (*n* = 3). Th, thalamus; HTh, hypothalamus; Ctx, cortex; Hp, hippocampus

In both i.p. and i.c. groups, individual brain regions responded to prions in a region-specific fashion (Fig. 3a). However, within individual brain regions, the vast majority of DEGs were common across four strains (Fig. 3b), whereas only a small fraction of DEGs exhibited strain-specific up- or downregulation. As expected, the most strain-specific DEGs were found in SSLOW, which is likely to reflect the fact that SSLOW-infected animals displayed the most severe degree of astrocyte activation. Combined GSA scores summated for four brain regions were used to report on the degree of astrocyte activation along with dysregulation in astrocyte physiological functions. Plotting of the combined GSA scores against the time to the terminal disease for all animals, which included i.c. and i.p groups, confirmed a very strong relationship between the degree of activation/dysregulation and the incubation time (Fig. 4a). In comparison to the correlation build using both i.c. and i.p. groups (R^2^ = 0.69), the strength of the relationship was even higher for the i.c. groups (R^2^ = 0.84), but weaker for the i.p groups (R^2^ = 0.64), (Fig. 4a–c). A weaker relationship in i.p. groups was expected, considering that upon i.p. challenges, the incubation time to diseases is dictated in part by the strain-dependent interaction of prions with peripheral cells and timing of invasion of CNS [28–33].

To investigate how the strain-specific regional tropism of prions affects the relationship between the changes in gene expression and time to terminal disease, we examined each brain region separately. Ctx and HTh showed the strongest correlation, whereas Hp (both i.c. and i.p. groups) and Th (i.c. group) showed weak correlations (Additional file 7: Fig. S2). Weak correlation in i.p. Hp groups was attributed to substantial strain-specific differences in tropism to Hp, which disturbed the relationship. The strength of the relationship for Hp and Th regions of the i.c. groups was also weak. Hp and Th were affected uniformly strong in all i.c.-infected groups, as evident from a narrow range of GSA scores in both regions and the heat map (Fig. 3a), which explained a weak relationship. Remarkably, summation of GSA scores across all four regions absorbed the disturbances due to strain-specific neurotropism (Fig. 4).

### Gene set changes that define the time to the terminal stage

**Fig. 5:**
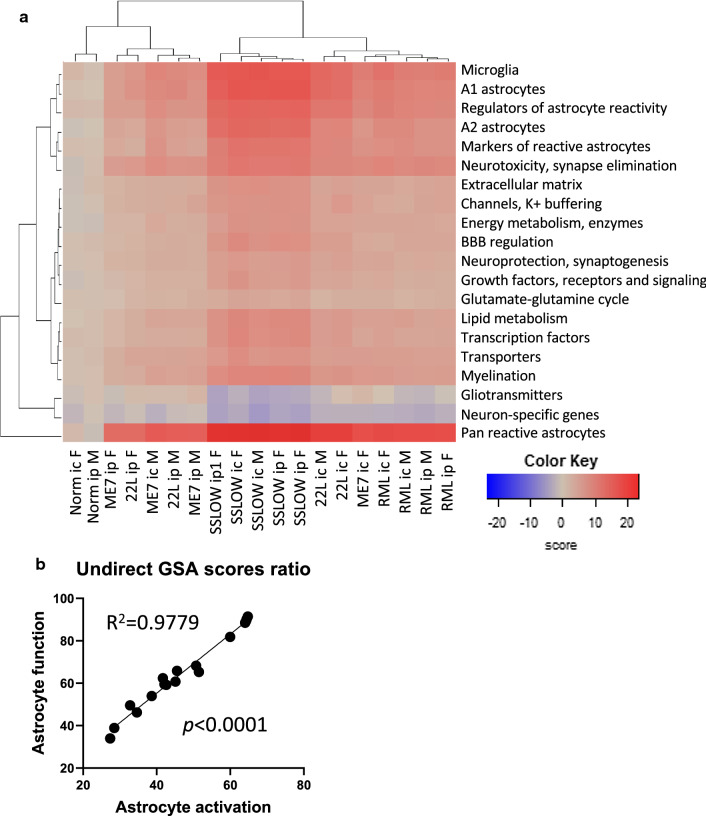
A relationship between the changes in the expression of gene sets in cortex. (**a**) Heat map of directed global significance scores for 20 gene sets across 19 groups of animal samples. (**b**) A strong correlation between the sum of undirected global significance scores of the gene sets reporting on the degree of astrocyte activation (A1-, A2-, pan-specific markers and other markers of reactive astrocytes) and dysregulation of astrocyte function (BBB regulation, channels, lipid/cholesterol homeostasis, myelination, energy metabolism, extracellular matrix, gliotransmitters, transporters, glutamate-glutamine cycle, neuroprotection, neurotoxicity)

Next, to evaluate the inverse correlation with the timing to disease on a gene set level, we looked at the GSA scores for individual astrocyte functions. The majority of gene sets were disturbed in all strains, although to a different extent (Fig. 5a). As expected, SSLOW groups, which had the shortest incubation times, clustered together and displayed the strongest changes in all gene sets. Notably, pathway-specific heat map analysis did not reveal a pathway responsible for the disease-associated phenotype, but instead pointed to a global disturbance of genes across multiple astrocyte-specific functions.

**Fig. 6:**
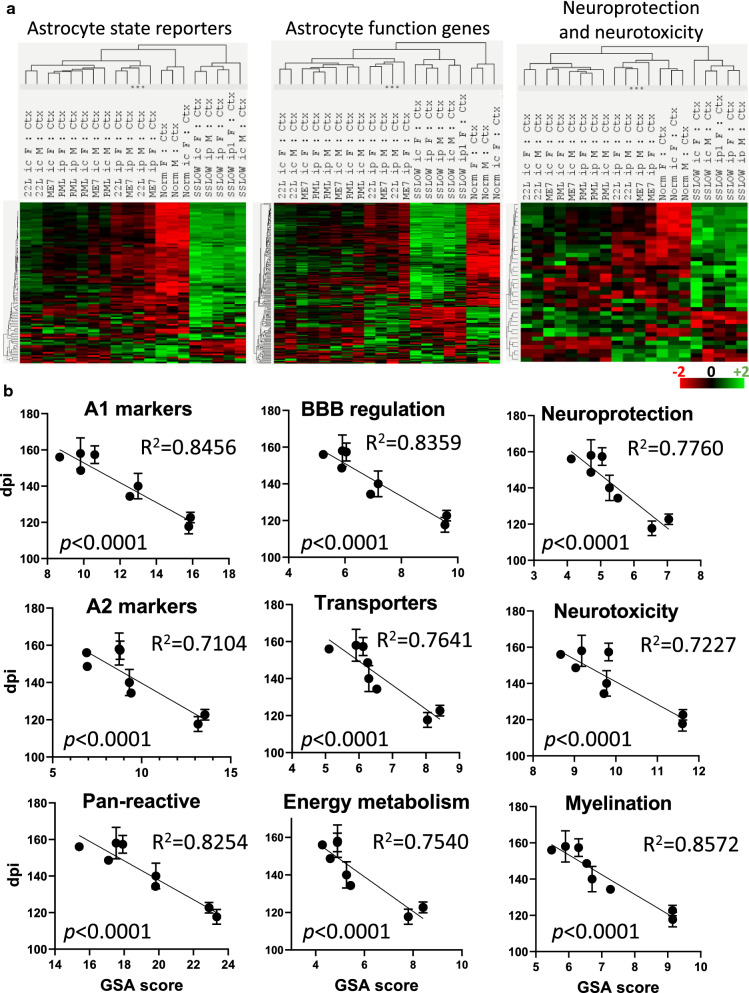
Correlations between GSA scores for individual gene sets and incubation time to disease. (**a**) Hierarchical clustering of grouped data for cortex (*n* = 6 for normal females, *n* = 3 for other groups). Astrocyte state reporters (left panel) include A1-, A2-, pan-specific markers, other markers of reactive astrocytes, regulators of astrocyte reactivity, transcription factors, growth factors, and receptors. Astrocyte function genes (middle panel) include BBB, channels, lipid/cholesterol homeostasis, myelination, energy metabolism, extracellular matrix, gliotransmitters, transporters, and glutamate-glutamine cycle genes. Genes related to the astrocyte function in neuroprotection/synaptogenesis, and neurotoxicity/synapse elimination are presented on the right panel. (**b**) Simple linear regressions built for GSA scores of selected gene sets and incubation time to disease showing a strong inverse correlation. Cortex samples from i.c. female and male groups were used

Examining individual gene sets based on the dataset of cortices in i.c. groups revealed pathways with the strongest relationship (R^2^ > 0.70) to the incubation time to disease. They included pathways reporting on the astrocytes reactive states or involved in regulating astrocyte reactivity (pan-, A1- and A2-specific markers; Other markers of reactive astrocytes; Regulation of astrocyte reactivity), as well as multiple pathways reporting on astrocyte function (BBB regulation, Transporters, Myelination, Energy metabolism, Channels, Extracellular matrix, Growth factors/receptors/signaling, Neuroprotection, Neurotoxicity, Transcription factors) (Fig. 6, Additional file 5: Table S5). Few remaining pathways showed relatively weak, yet statistically significant, strength of the relationship (Additional file 3: Table S3). Weaker strength might not necessarily reflect low impact of the remaining pathways, but can be attributed, in part, to a small size of a particular gene set in the Astrocyte panel. In fact, the top 40 differentially expressed functional pathway genes included multiple genes from gene sets with a weak relationship to incubation time (Additional file 6: Table S6). Notably, the degree of astrocyte activation, as measured by upregulation of pan-, A1- and A2-markers, and the severity of dysregulation in physiological pathways were tightly related. Indeed, there was a very strong correlation coefficient (R^2^ = 0.98) between the gene sets reporting on the degree of activation and the dysregulation in physiological pathways (Fig. 5b).

**Fig. 7:**
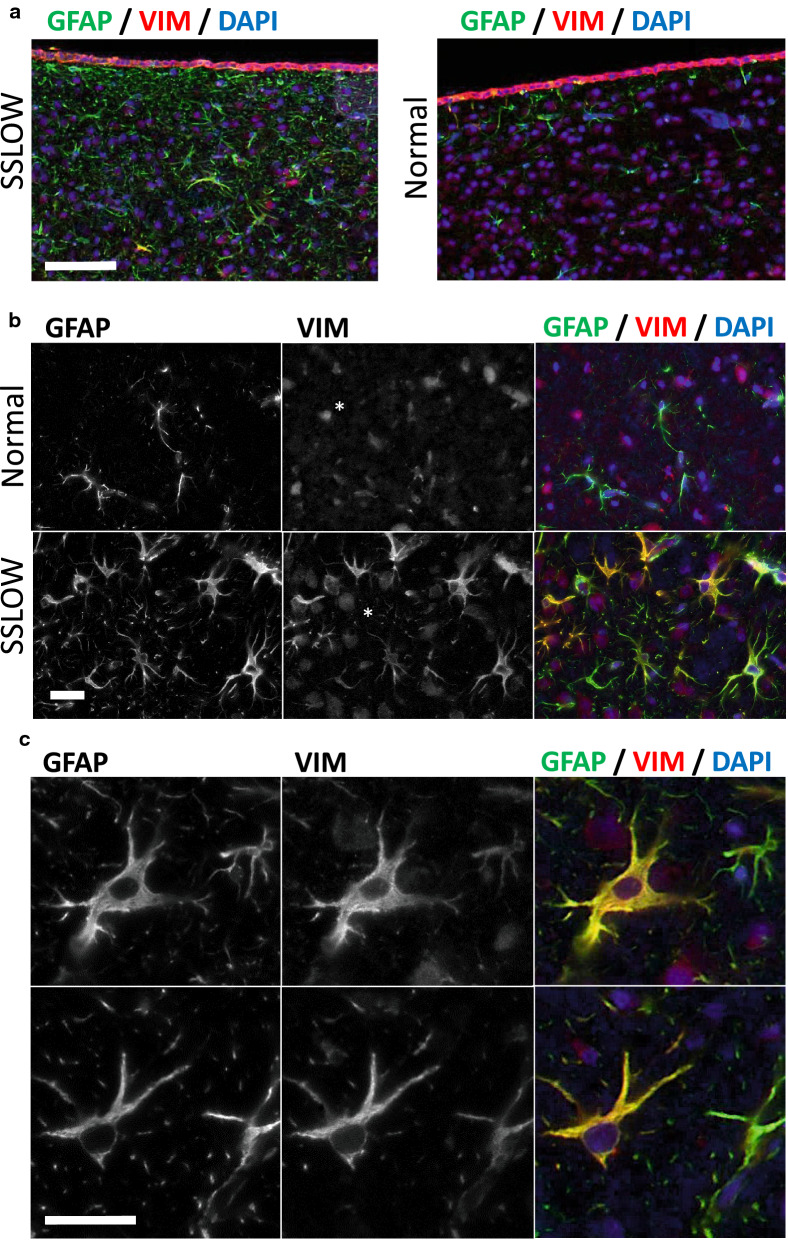
Immunoreactivity of vimentin in SSLOW-infected animals. Co-immunostaining of SSLOW-infected mice and age-matched control for vimentin (VIM, red), GFAP (green) and nuclei (DAPI, blue). (**a**) Similar to control mice (right), SSLOW-infected mice (left) maintain strong Vimentin immunoreactivity of ependymal cells. (**b, c**) Astrocytes in normal animals lacked vimentin immunoreactivity (**b**, top). In SSLOW-infected animals, hypertrophic GFAP + astrocytes consisted of heterogeneous VIM^+^ and VIM^−^ sub-populations (**b**, bottom and **c**). Scale bar: 60 μm in **a**, 20 µm in **b** and **c**. Asterisks: non-specific binding of anti-Vim antibodies to nuclei

**Fig. 8:**
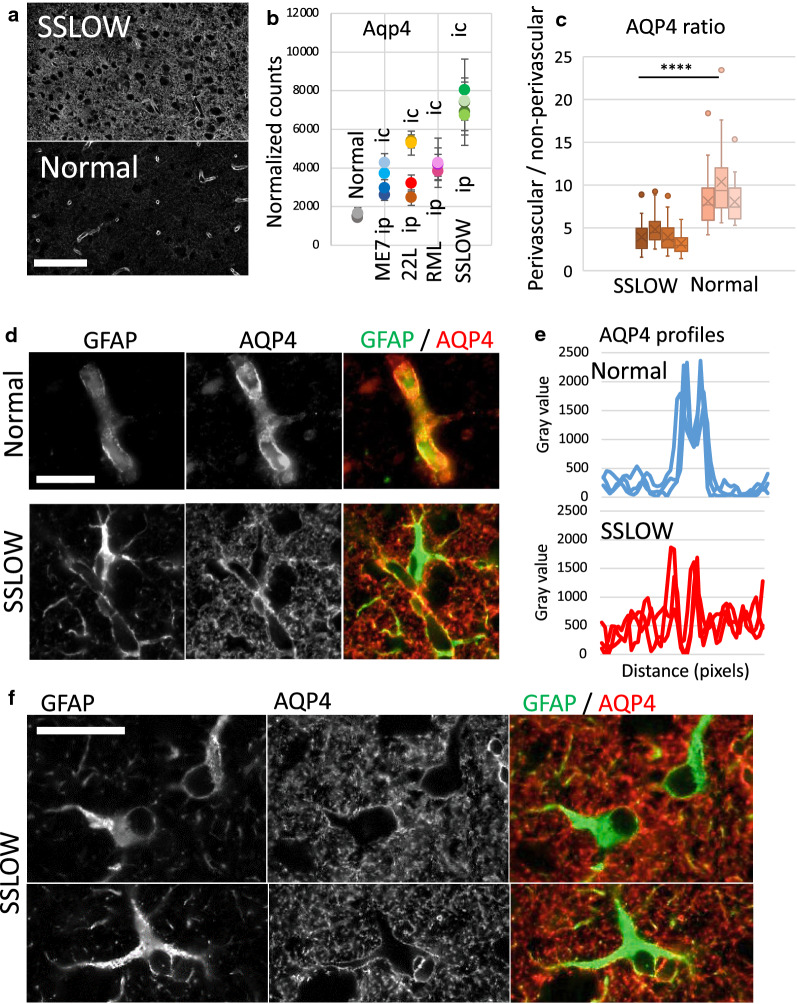
Change in cellular localization of AQP4 in prion-infected mice. (**a**) Reduction of perivascular AQP4 and increase of non-perivascular AQP4 in the cortex of SSLOW-infected mice (top) in comparison to age-matched control mice (bottom). (**b**) Normalized count of AQP4 mRNA in cortex plotted for different groups of mice. NanoString data were plotted as group averages with standard deviations (*n* = 6 for normal females, and *n* = 3 for other groups). (**c**) Ratio of perivascular versus non-perivascular AQP4 immunofluorescence signal in cortex of SSLOW and age-matched control mice. (**d**) Higher magnification images of cortical vessels in age-matched control (upper row) and SSLOW-infected mice (lower row) co-immunostained for GFAP (green) and AQP4 (red). (**e**) Representative profiles across microvessels in the cortices of age-matched control (upper plot) and SSLOW-infected mice (lower plot). (**f**) AQP4 immunoreactivity outlines hypertrophic astrocytes of SSLOW-infected mice. Scale bar 60 µm in **a**, 20 µm in **d** and **f**

For validating changes in gene expression, Vim, a pan-reactive marker expressed in disease-associated astrocytes in mouse models of Alzheimer’s disease [22], and Aqp4, the most prevalent water channel in CNS, were selected. In a normal brain, Vim was expressed predominantly in endothelial cells (Fig. 7a). In prion-infected animals, we observed an increase in Vim immunoreactivity, which colocalized with GFAP-positive astrocytes (Fig. 7b, c). Consistent with previous studies that documented heterogeneity of astrocytes in neurodegenerative conditions, including heterogeneity in Vim expression [22], several sub-populations of GFAP^+^ astrocytes ranging from Vim negative to strongly Vim positive were seen in prion-infected brains (Fig. 7b, c). Immunostaining for Aqp4 revealed that upregulation in Aqp4 expression was accompanied by remarkable changes in its subcellular colocalization (Fig. 8). In normal brains, Aqp4 was found predominantly in astrocytic end-feet that surround microvessels, whereas in prion-infected animals, intense Aqp4 reactivity was widespread in processes (Fig. 8a, c-f). In fact, changes of the ratio of perivascular versus non-perivascular Aqp4 immunoreactivity documented a remarkable sub-cellular redistribution of this channel protein in reactive astrocytes. These changes were reminiscent of those seen in Creutzfeldt-Jakob disease individuals [34].

### Changes in neuroprotective versus neurotoxic gene sets

Both sets of genes, involved in neuroprotection and neurotoxicity, were among the groups with a strong relationship to the incubation time (Fig. 6). In fact, plotting GSA scores for neuroprotective versus neurotoxic pathways revealed a very strong relationship between the two gene sets (R^2^ = 0.95), suggesting that in reactive astrocytes, both sets change in parallel (Additional file 7: Fig. S4). Regardless of strain or brain region, the majority of DEGs involved in neurotoxicity were upregulated, whereas a significant fraction of genes responsible for neuroprotection or synapse maintenance were downregulated (Additional file 7: Fig. S3, S4). Among the neuroprotective genes downregulated across four prion strains and four brain regions were neurexin 1 (*Nrxn1*) and neuroligin 1 (*Nlgn1*), the cell surface proteins that are required for neurotransmission by mediating formation and maintenance of synapses [35, 36]. Several neuroprotective genes were downregulated in a region-specific manner, including cadherin 10 (*Cdh10*), which is involved in maintenance of excitatory and inhibitory synaptic structures [37], and glypicans 4 and 5 (*Gpc4, Gpc5*), which are important for functional synapse formation [38]. While the transition to the reactive state underlies disturbances in both neuroprotective and neurotoxic gene sets, the net effect was consistent with a neurotoxic phenotype.

## Discussion

In a healthy brain, astrocytes are responsible for a number of important physiological functions including support of neuronal growth, modulation of neurotransmission, formation and maintenance of synapses, regulation of blood flow, supplying energy and providing metabolic support to neurons, maintaining the blood–brain-barrier and more [39–41]. In neurodegenerative diseases including prion diseases, astrocytes acquire reactive phenotypes sustained throughout the disease progression [1, 2, 42]. In recent studies on region-specific analysis of gene expression, astrocytes responded to prions much earlier than neurons and even sooner than microglia, and astrocyte functions scored at the top of the activated pathways [13]. Analysis of hippocampal proteome revealed a predominant astrocytic signature in prion-infected mice [43]. With a growing appreciation of the role of astrocytes in neurodegenerative diseases, the questions whether astrocytes lose their ability to perform normal physiological functions in the reactive states and whether the reactive phenotypes are neurotoxic or neuroprotective still remain unsettled [2, 12, 43, 44]. These questions are closely related to another important query—do astrocytes simply respond to altered brain homeostasis or, on the contrary, drive pathogenesis?

For addressing the above questions, the current work examined animals challenged with four prion strains via two inoculation routes using a gene panel that monitored expression of astrocyte-specific genes. Under the circumstances where astrocytes simply respond to changes in the CNS environment, one should expect more severe changes in groups with longer incubation times, due to longer exposure to altered brain homeostasis along with a more pronounced impact of natural aging. Indeed, in normal aged animals, dysregulation in astrocytic gene expression was found to be reminiscent of those observed in neurodegenerative diseases [45–47]. Moreover, astrocytes primed by chronic neurodegeneration were found to produce an exaggerated, pro-inflammatory response [48]. In the current study, a reverse correlation between the degree of astrocyte activation and the incubation time to the diseases was observed. SSLOW-infected animals had the shortest incubation times and the most severe astrocyte response, whereas the most attenuated response was observed in ME7 females with the longest incubation time to the disease. The strength of the relationship, as measured by the correlation coefficient, was very high for the i.c. groups (R^2^ = 0.84), lower for the combined i.c. and i.p. groups (R^2^ = 0.69), and the lowest, yet still robust and highly significant, for the i.p. groups (R^2^ = 0.62). Strain-specific differences in pathogenic steps that preceded neuroinvasion such as interaction of prions with peripheral immune cells, replication in secondary lymphoid organs and trafficking to the CNS [28–33], were likely to disturb the strength of correlation between astrocytic response and the incubation time to the terminal disease upon peripheral challenges. Nevertheless, the current work suggests that the degree of astrocyte activation along with dysregulation of their physiological pathways are major contributors in defining the timeline of disease progression.

As illustrated by a region-specific transcriptome profile (Fig. 1), astrocytes exhibit robust regional homeostatic identity, while responding to prion infection in a region-specific manner [42, 49]. Individual prion strains follow their own, strain-specific timelines of disease progression exhibiting strain-specific affinity to different brain regions [13, 25, 50]. It is not surprising that, in individual brain regions, the relationships between astrocyte response and incubation time were not as strong as the relationship build based on the combined scores across four brain regions. Remarkably, scores combined across four regions absorbed disturbances attributed to the region-specific differences in strain tropism and region-specific astrocyte response.

The four prion strains used in the current work exhibit different cell-specific tropism. 22L is mainly associated with astrocytes, ME7 is primarily found in neurons, SSLOW is colocalized with microglia and considerably less with astrocytes, whereas cell association of RML depends on brain region, showing either neuron- or astrocyte-specific localization [19, 24]. Regardless of the differences in the cellular tropism, the set of DEGs largely overlapped between all four strains, displaying very minor strain-specific differences (Fig. 3b). In part, strain-specific DEGs can be explained by differences in the degree of astrocyte activation, as was clearly the case for SSLOW, which exhibited the largest number of strain-specific DEGs and the most severe degree of astrocyte activation. These results suggest that the astrocyte reactive phenotype, along with the nature of dysregulation in their physiological pathways, were universal and not dictated by cell type, with which individual prion strains were associated.

What normal functions of astrocytes are disturbed in prion diseases? The fold changes for the individual functional genes, including the top 40 functional genes (Additional file 4: Table S4), were not as dramatic as the genes that reported on astrocyte reactive phenotypes. Nevertheless, plotting the GSA scores for individual groups of genes clearly showed strong inverse relationships between the changes in gene sets and the time to terminal disease. Pathways that report on a number of physiological functions including BBB regulation, transporters, myelination, energy metabolism, channels, extracellular matrix, growth factors/receptors/signaling, neuroprotection, neurotoxicity and transcription factors showed very strong relationships with correlation coefficients ranging between 0.7 and 0.86. For the remaining gene sets, the correlation coefficients were lower, but also suggested statistically significant correlations. Because individual functions were represented only by limited numbers of genes, the correlation coefficient might not report on the actual impact of the individual pathways. This work suggests that the manifestation of the reactive states associated with prion diseases was not in dysfunction of any specific pathway, but a global transformation of the physiological state of astrocytes, characterized by disturbance in multiple functions. Notably, the more substantial the disturbance, the shorter the incubation time.

Analysis of gene expression alone might not report on the depth of functional transformation in astrocytes to the full extent. Dramatic changes in cellular localization of AQP4 provided an excellent illustration of this point. In the current work, upregulation of AQP4 ranged from ~ 1.5- to fourfold, an observation consistent with the previously reported increase in expression levels of AQP4 in prion diseases of animals and humans [34, 51–53]. Changes in the cellular localization of AQP4 from predominantly end-feet, which surround microvessels, to cell processes, which lack direct contact with blood vessels, supported the hypothesis regarding profound transformation in astrocyte physiology. AQP4 is the most prevalent, bidirectional water channel in the CNS. Under normal conditions, AQP4 localized in astrocytic end-feet and is considered a key channel in maintaining water homeostasis [54]. In addition, AQP4 is a critical part of the glymphatic system responsible for removing neuronal wastes including abnormal tau and Aβ peptides [55, 56]. Since minor changes in water homeostasis perturb ion concentrations and neuronal excitability [57], changes in sub-cellular localization of AQP4 are expected to have profound effects in physiology of the CNS including impact on glymphatic drainage.

Changes in functional states of astrocytes occurred in parallel with the transformation in their reactive state. According to the Barres hypothesis, astrocytes can polarize and acquire either neurotoxic (A1) or neuroprotective (A2) phenotypes, which exhibit distinct transcriptome characteristics and, presumably, opposing effects on neuronal survival [58, 59]. In the current work, a number of A1-, A2- and pan-reactive markers were upregulated, where the level of upregulation within each group correlated strongly with the incubation times (R^2^ = 0.85 for A1-, R^2^ = 0.71 for A2- and R^2^ = 0.83 for pan-reactive markers). These results are in agreement with previous studies [12, 13, 16, 19, 44] and illustrate that in prion diseases, astrocytes do not follow the simple A1/A2 polarization model. The concept for polarization into A1 or A2 states was developed using animals treated with LPS or subjected to ischemia (MCAO), conditions that did not induce sustainable, chronic effects [58]. The current work suggests that in actual chronic neurodegenerative disease, the polarization phenotype is complex and involves upregulation of markers previously assigned to neurotoxic (A1) and neuroprotective (A2) phenotypes along with pan-reactive markers. The current work illustrates that the reactive phenotype associated with prion diseases is characterized by dysregulation in a number of physiologically important functions.

In the current work, mRNA expression was analyzed in bulk tissues opening possibilities that changes in gene expression were attributed not only to astrocytes, but also to other cell types. Indeed, despite the fact that pan-, A1- and A2-markers have been used widely to describe the reactive state of astrocytes, the majority of these markers along with genes responsible for neuroinflammation are known to be expressed by multiple cell types [44]. The possibility that under the disease conditions, multiple cell types contribute to differential expression of genes associated with astrocyte-specific physiological pathways, cannot be excluded. If this is the case, dysregulation of astrocyte-specific pathways described in this study involves, at least in part, a cell-non autonomous mechanism.

Previous studies that employed single-cell RNA sequencing established that astrocytes not only display region-specific homeostatic signatures, but also exhibit subregional heterogeneity defined by developmental patterns [20–22, 60]. In mouse models of Alzheimer’s disease or individuals with Alzheimer’s disease, in addition to the sub-populations that constitute a healthy brain, a new sub-population with distinctive transcriptome signatures termed disease-associated astrocytes (DAAs) emerged [22]. VIM, which is in a normal brains expressed predominantly by endothelial and ependymal cells, was identified as one of the main markers that constitute the disease-associated signature in astrocytes [22]. The current study analyzed bulk tissues and could not identify sub-populations of astrocytes with distinctive transcriptome signatures. Nevertheless, consistent with the work on single cell RNAseq, diverse populations of astrocytes were seen in the current work including GFAP^+^VIM^−^, GFAP^+^VIM^+^ and GFAP^+^ astrocytes with low levels of VIM expression. We do not know, whether heterogeneity in VIM expression levels represent different degrees of astrocyte activation, different sub-populations of disease-associated astrocytes, normal vs disease-associated subpopulations or all of the above. It will be interesting to examine in future studies whether reactive astrocytes associated with prion diseases are shared with other neurodegenerative diseases or prion-specific.

The current work suggests that phenotypic changes in the state of astrocytes contribute to the faster progression of diseases and perhaps even drive prion pathogenesis. Notably, both sets of genes, those involved in neuroprotection and neurotoxic functions, were disturbed in the reactive astrocytes. It appears that in the reactive states, astrocytes upregulate a group of genes associated with neuronal protection, which is in agreement with previous work documenting upregulation of defense pathways against protein misfolding and reactive oxygen species [43]. In parallel, several important genes involved in formation and maintenance of synapses (*Nrxn1*, *Nlgn1, Cdh10, Gpc4, Gpc5*) were found to be downregulated. While some neuroprotective pathways might be upregulated in response to prions, the net result of disturbances in neuroprotective/neurotoxic pathways, along with the global dysregulation across physiological functions, produced a neurotoxic phenotype. Indeed, several recent studies argue that reactive astrocytes associated with prion diseases are neurotoxic. Primary astrocytes isolated from prion-infected animals had adverse effects on neuronal cultures invoking reduction in spine size and density along with impairment of neuronal growth and synapse integrity [15]. Synaptotoxic effects of primary reactive astrocytes were mediated via astrocyte-conditioned media, as well as also seen in astrocyte-neuron co-cultures [15]. Selective, astrocyte-restrictive targeting of unfolded protein response, which is exuberated in reactive astrocytes, via inhibition of PERK signaling was found to prolong the incubation time to the terminal disease in mice [14]. Moreover, highly infectious prions lacked direct neurotoxicity, indirectly supporting the hypothesis on a non-cell autonomous mechanism of neurodegeneration [17]. Together with previous work, the current study argues in support of the non-cell autonomous, astrocyte-driven mechanism behind neurodegeneration.

What factors dictate the incubation time to the diseases? Physical properties of prions including strain-specific replication rate, conformational stability and size of protein aggregates were examined in the past in attempt to establish a link between strain-specific characteristics and disease progression [61–64]. The current work suggests that the degree of astrocyte activation and dysregulation in their physiological functions dictate the rate of disease progression. It is not clear how astrocytes are activated in prion diseases, whether direct PrP^Sc^-astrocyte interaction is important for their activation, and to what molecular features of PrP^Sc^, if any, astrocytes respond. Recent studies that employed triple TNF^−/−^/IL1a^−/−^/C1qa^−/−^ knockout mice questioned the role of reactive microglia as a driver of the neurotoxic phenotypes in astrocytes [12]. Elimination of three key microglia-derived factors TNF-α, IL-1α and C1qa, which are known to drive the A1 reactive state, was found to have only modest effects in suppressing A1-specific markers in prion-infected animals and, contrary to the expectations, accelerated the progression of prion diseases [12]. Moreover, partial ablation of microglia by PLX5622 exacerbated the reactive astrocyte phenotype and accelerated disease progression, suggesting that in prion diseases, astrocyte activation does not rely on microglia, but in contrast is attenuated by reactive microglia [16]. In neurons, lipoprotein receptor-related protein 1, a transmembrane protein which is also expressed in astrocytes at high levels, was shown to be involved in recognition and endocytosis of PrP^Sc^ [65]. In previous studies, prion protein fragment PrP 106–126 induced hypertrophic changes along with upregulation of GFAP expression in astrocytes cultured in vitro [66]. In mice infected with prions via i.c. or i.p routes, the onset of GFAP upregulation appeared to be triggered by the accumulation of PrP^Sc^ over a certain threshold, and the kinetics in GFAP overexpression followed very closely the kinetics of PrP^Sc^ accumulation [67]. Furthermore, previous work also revealed that cultured astrocytes could recognize PrP^Sc^ directly and, in response, upregulate expression of chemokine genes triggering microglia migration [68]. Microglia too can recognize and react to PrP^Sc^ with a pro-inflammatory response, where the degree of the response was found to be dictated by the sialylation status of N-linked glycans on the surface of PrP^Sc^ [69]. Interestingly, the level of PrP^Sc^ sialylation varies among prion strains [32, 70]. Notably, significant changes in PrP^Sc^ sialylation pattern accompanied the shortening of the incubation times to the disease during serial adaptation of a prion strain to a new species, suggesting that the PrP^Sc^ sialylation pattern dictate glial activation and disease progression [19, 71]. In conclusion, the current work supports the view of reactive astrocytes as emerging drivers of pathogenesis in prion disease. This opens new venues for developing new therapeutic targets that aim to manipulate the reactive states of astrocytes.

## Supplementary Information

**Additional file 1. Table S1**: Composition of nCounter Astrocyte panel. Gray font color and asterisks indicate probes producing low counts.

**Additional file 2. Table S2**: List of animal groups analyzed using the Astrocyte panel.

**Additional file 3. Table S3**: Normalized counts for all individual animals listed in Table S2, reported for four brain regions.

**Additional file 4. Table S4** Linear fold change for each gene, reported as a ratio between each sample group and normal i.p. female (F) as a control group. The table includes data for four brain regions, presented as full lists of genes and as lists of changed genes (*p* < 0.05, fold change ≥-/+1.2).

**Additional file 5. Table S5**: List of gene sets in Astrocyte panel. Correlations (R2, p-value) between individual gene sets and incubation time to terminal disease calculated separately for four brain regions for i.c. and i.p. groups.

**Additional file 6. Table S6**: Top 40 DEGs in pathways that report on astrocyte function. Fold change along with p-values of the top 40 DEGs expressed in thalamus of all animal groups.

**Additional file 7.**:** Figure S1**. GFAP in normal and infected brains. (a) GFAP immunoreactivity in cortex, hippocampus and thalamus of normal mice and of mice inoculated i.p. with 22L or SSLOW. Scale bar 100 μm. (b) Normalized GFAP counts detected by NanoString in cortex, hippocampus and thalamus of normal mice and of mice inoculated i.p. with 22L or SSLOW (*n* = 3).** Figure S2**. Linear regression for the combined GSA scores in four brain regions. Inverse correlations between the combined GSA scores and the time to terminal prion disease in cortex (Ctx), hippocampus (Hp), thalamus (Th) and hypothalamus (HTh) of i.p. and i.c. sample groups. ** Figure S3**. DEGs in neurotoxicity and neuroprotection gene sets in four prion strains. (a) Log2 fold changes in neurotoxicity and neuroprotection gene sets in thalamus of male mice i.c.-inoculated with ME7, 22L, RML, or SSLOW (n=3; * p < 0.05, ** p < 0.01, *** p < 0.001, **** p < 0.0001). (b) Correlation between undirected global significance scores of neurotoxicity and neuroprotection gene sets calculated for thalamus of female and male groups inoculated via i.p. and i.c. routes.**Figure S4**. DEGs in neurotoxicity and neuroprotection gene sets in four brain regions. Log2 fold changes in neurotoxicity and neuroprotection gene sets in cortex (Ctx), hippocampus (Hp), thalamus (Th) and hypothalamus (HTh) of female mice i.c.-inoculated with SSLOW (n=3; * * p* < 0.05, ** * p* < 0.01, *** * p* < 0.001, **** p < 0.0001).

## Data Availability

All data generated or analyzed during this study are included in this published article and its supplementary information file.

## Abbreviations

22L: Mouse adapted prion strain
ME7: Mouse adapted prion strain
RML: Mouse adapted prion strain
SSLOW: Mouse adapted prion strain
A1: Reactive state of astrocytes
A2: Reactive state of astrocyte
AQP4: Aquaporin 4
BBB: Blood brain barrier
DGEs: Differentially expressed genes
GFAP: Glial fibrillary acidic protein
GSA: Gene set analysis
i.c.: Intracranial route
i.p.: Intraperitoneal route
Ctx: Cortex
Hp: Hippocampus
Hth: Hypothalamus
Th: Thalamus
PrP^Sc^: Infectious, disease-associated, pathogenic form of the prion protein
