# Supplementary material for: The degree of astrocyte activation is predictive of the incubation time to prion disease
Source: Acta Neuropathol Commun. 2021 May 12;9:87. doi: 10.1186/s40478-021-01192-9 (PMC8114720; doi:10.1186/s40478-021-01192-9)
Supplement: Supplementary file 2 — Additional file 2. Table S2: List of animal groups analyzed using the Astrocyte panel. [file 40478_2021_1192_MOESM2_ESM.pdf]

Table S2. List of animal groups analyzed using Astrocyte panel

Animal groups	Inoculation route	Inoculum, % [1]	n	Days post inoculation (dpi) at euthanasia
ME7 F	i.p.	1% SBH	3	295, 346, 363
ME7 M	i.p.	1% SBH	3	258, 272, 272
22L F	i.p.	1% SBH	3	168, 179, 225
22L M	i.p.	1% SBH	3	197, 203, 225
RML F	i.p.	1% SBH	3	194, 205, 205
RML M	i.p.	1% SBH	3	212, 219, 224
SSLOW F ip1	i.p.	1% SBH	3	166, 173, 173
SSLOW F	i.p.	10% SBH	3	142, 146, 147
SSLOW M	i.p.	10% SBH	3	146, 155, 156
ME7 F	i.c.	10% SBH	3	147, 148, 151
ME7 M	i.c.	10% SBH	3	156, 156, 156
22L F	i.c.	10% SBH	3	133, 135, 135
22L M	i.c.	10% SBH	3	135, 137, 148
RML F	i.c.	10% SBH	3	154, 155, 163
RML M	i.c.	10% SBH	3	148, 163, 163
SSLOW F	i.c.	10% SBH	3	121, 121, 126
SSLOW M	i.c.	10% SBH	3	113, 120, 120
Norm F	i.p.	1xPBS	6	197, 223, 223, 295, 346, 363
Norm M	i.p.	1xPBS	3	203, 225, 229
Norm i.c. F	i.c.	10% NBH	3	137, 137, 137

[1] SBH – scrapie brain homogenate; PBS – phosphate-buffered saline; NBH – normal brain homogenate.
