# Supplementary figures and images for: The degree of astrocyte activation is predictive of the incubation time to prion disease
Source: Acta Neuropathol Commun. 2021 May 12;9:87. doi: 10.1186/s40478-021-01192-9 (PMC8114720; doi:10.1186/s40478-021-01192-9)

Figure S1

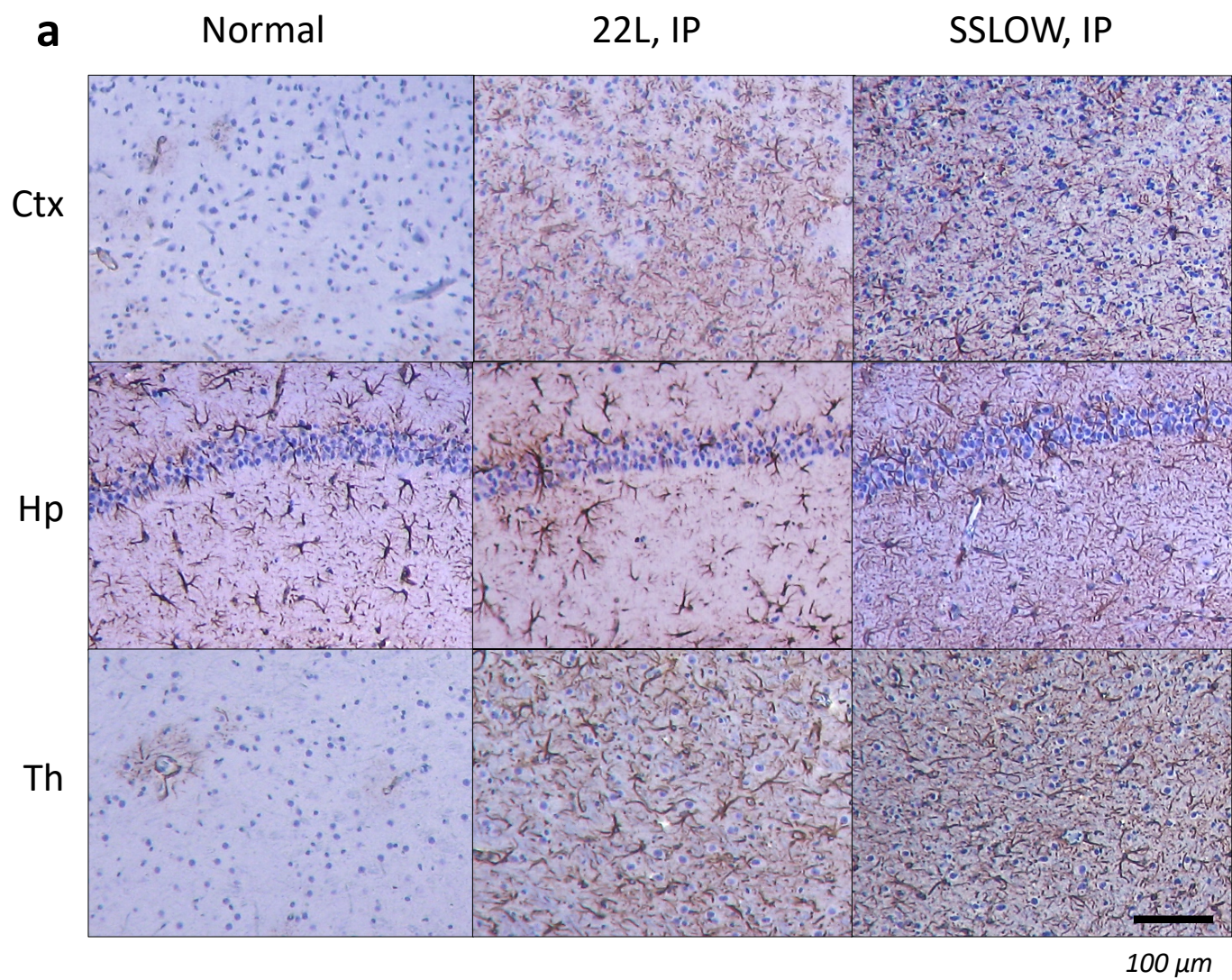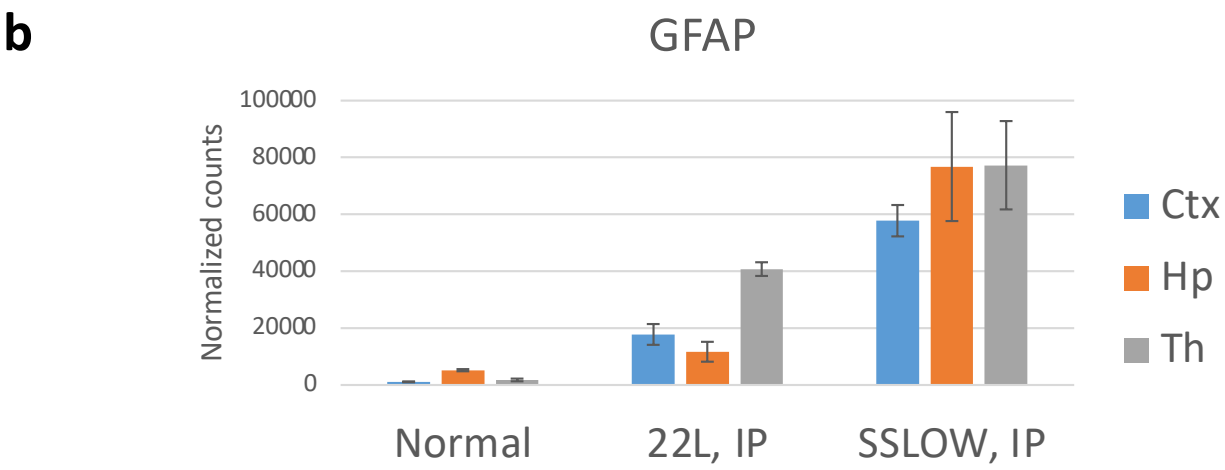

Figure S2

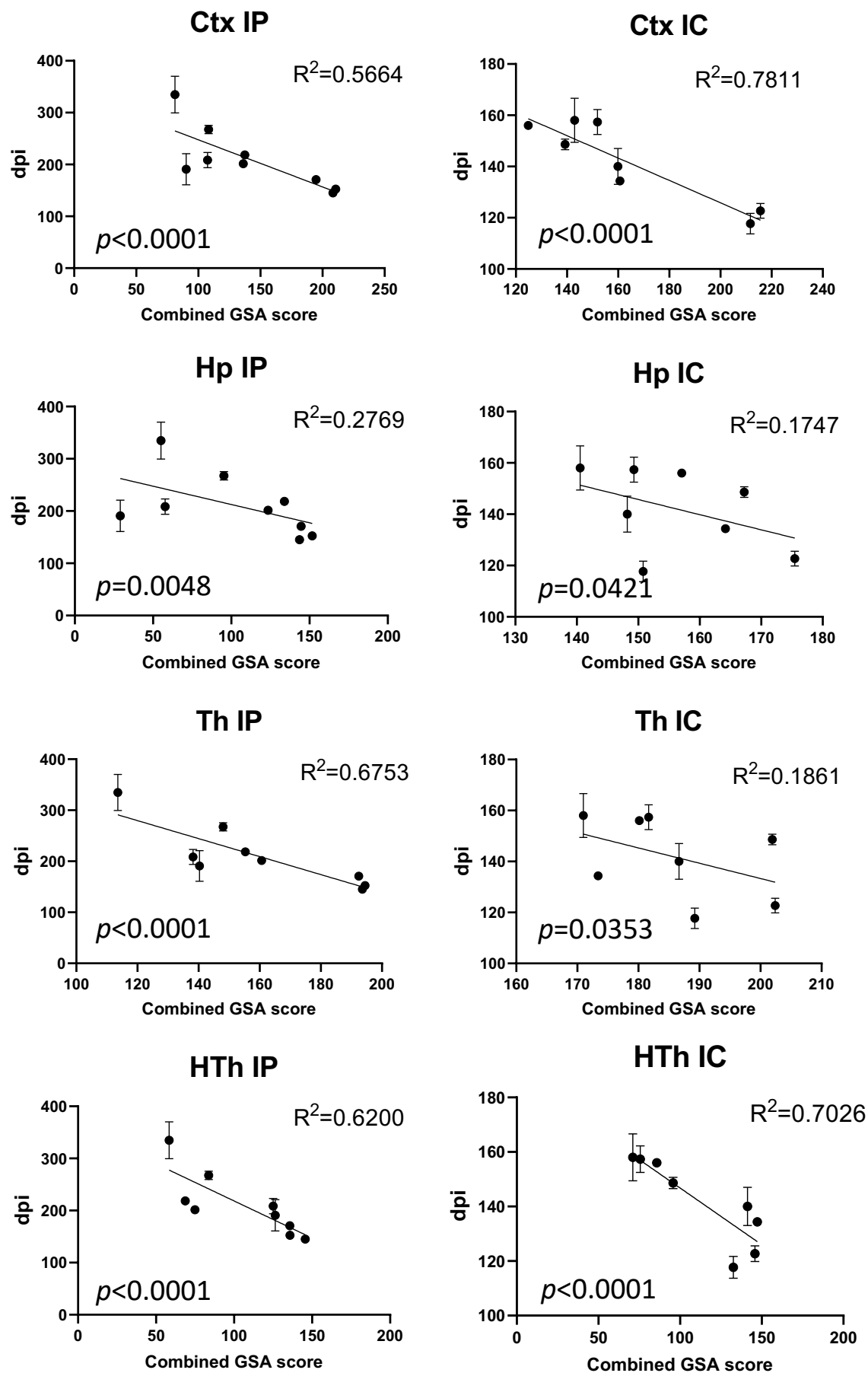

Figure S3

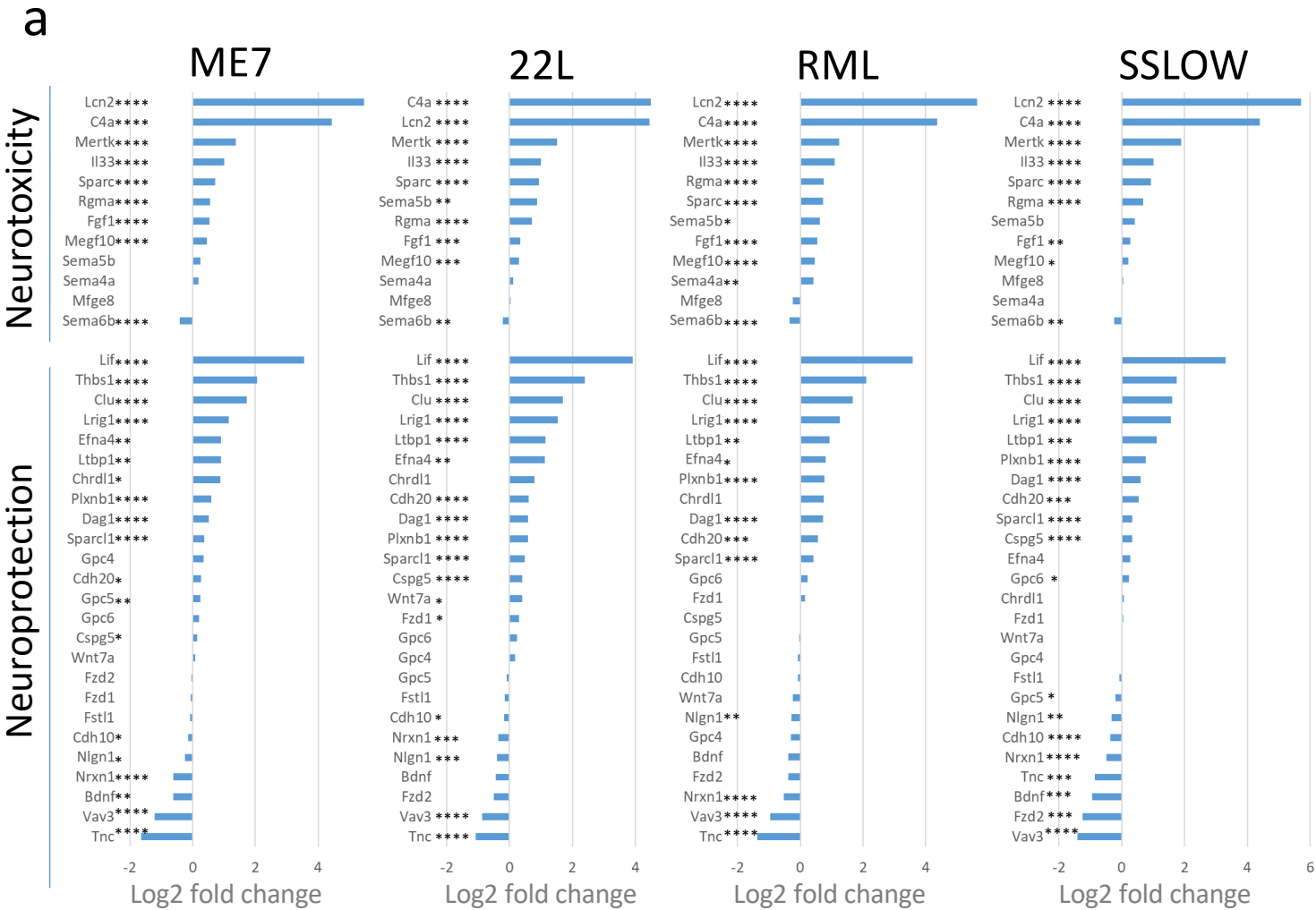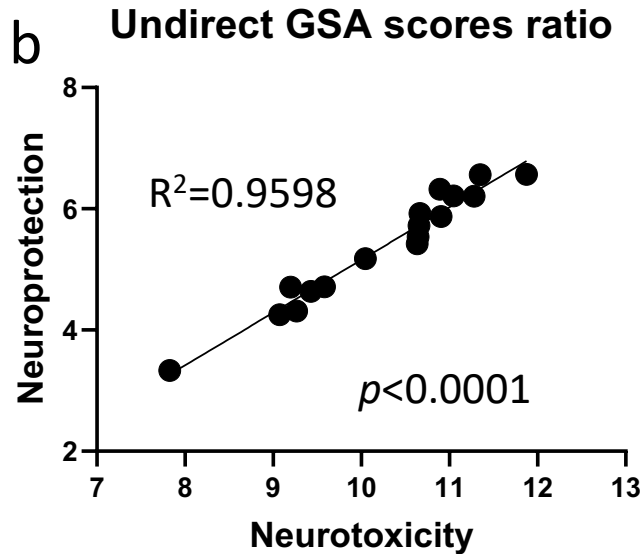

Figure S4

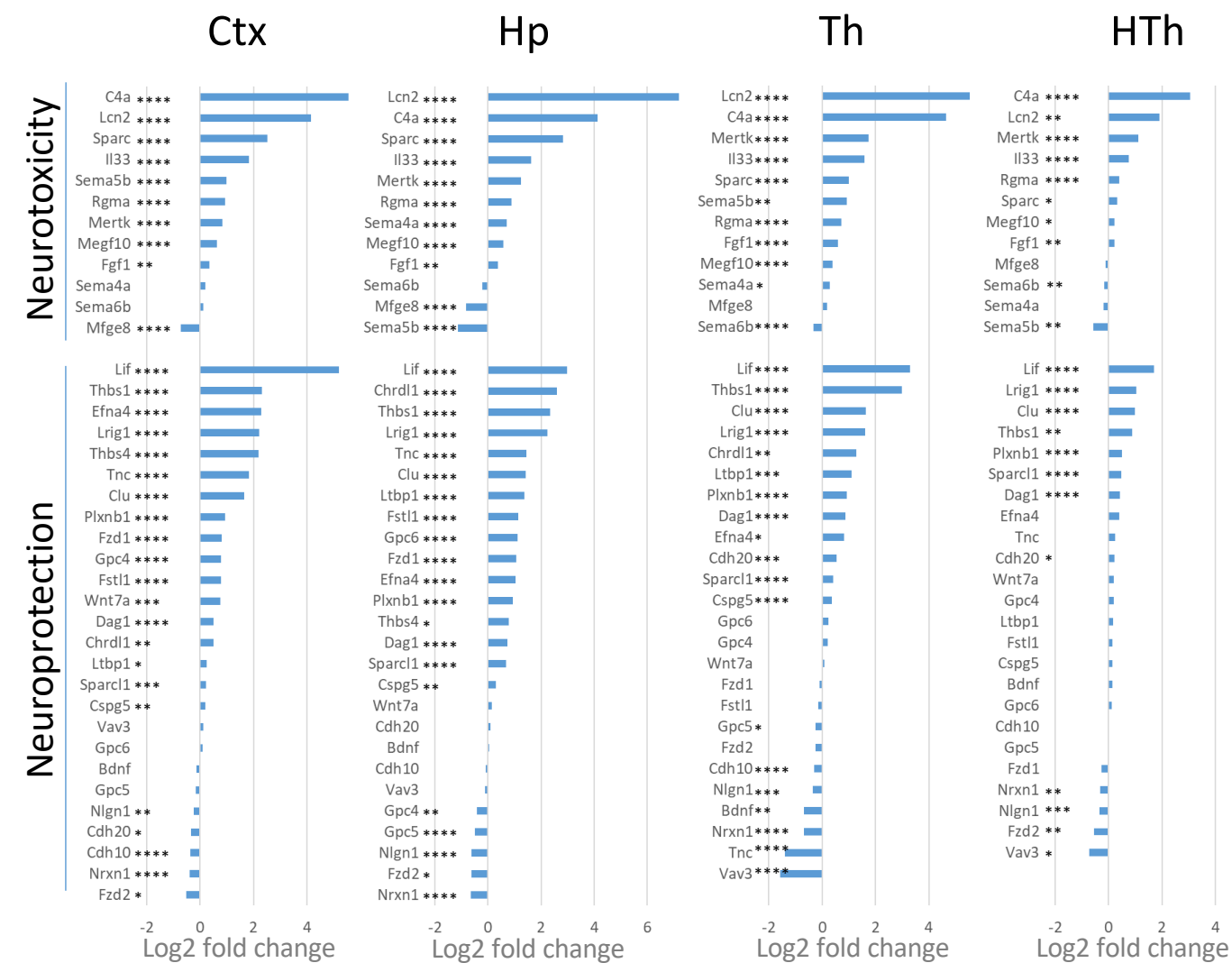

Supplement: Supplementary file 7 — Additional file 7.: Figure S1. GFAP in normal and infected brains. (a) GFAP immunoreactivity in cortex, hippocampus and thalamus of normal mice and of mice inoculated i.p. with 22L or SSLOW. Scale bar 100 μm. (b) Normalized GFAP counts detected by NanoString in cortex, hippocampus and thalamus of normal mice and of mice inoculated i.p. with 22L or SSLOW (n = 3). Figure S2. Linear regression for the combined GSA scores in four brain regions. Inverse correlations between the combined GSA scores and the time to terminal prion disease in cortex (Ctx), hippocampus (Hp), thalamus (Th) and hypothalamus (HTh) of i.p. and i.c. sample groups. Figure S3. DEGs in neurotoxicity and neuroprotection gene sets in four prion strains. (a) Log2 fold changes in neurotoxicity and neuroprotection gene sets in thalamus of male mice i.c.-inoculated with ME7, 22L, RML, or SSLOW (n=3; * p < 0.05, ** p < 0.01, *** p < 0.001, **** p < 0.0001). (b) Correlation between undirected global significance scores of neurotoxicity and neuroprotection gene sets calculated for thalamus of female and male groups inoculated via i.p. and i.c. routes.Figure S4. DEGs in neurotoxicity and neuroprotection gene sets in four brain regions. Log2 fold changes in neurotoxicity and neuroprotection gene sets in cortex (Ctx), hippocampus (Hp), thalamus (Th) and hypothalamus (HTh) of female mice i.c.-inoculated with SSLOW (n=3; * p < 0.05, ** p < 0.01, *** p < 0.001, **** p < 0.0001). [file 40478_2021_1192_MOESM7_ESM.pdf]
